# Small intestinal microbiota of undernourished women of reproductive age and microbiota-directed balanced energy protein (MD-BEP) supplementation in maternal environmental enteric dysfunction (EED): protocol for a community-based intervention study

**DOI:** 10.1186/s12884-026-08710-4

**Published:** 2026-02-06

**Authors:** Md. Shabab Hossain, Mustafa Mahfuz, M. Masudur Rahman, S.M. Khodeza Nahar Begum, Shafiqul Alam Sarker, Tahmeed Ahmed

**Affiliations:** 1 Nutrition Research Division (NRD), International Centre for Diarrhoeal Disease Research, Bangladesh (icddr,b), Dhaka, Bangladesh; 2 Department of Gastroenterology, Manikganj Medical College, Dhaka, Bangladesh; 3 Department of Gastrointestinal, Hepatobiliary and Pancreatic Disorders, Bangladesh Specialized Hospital, Dhaka, Bangladesh; 4 Department of Histopathology & Cytology, Bangladesh Specialized Hospital, Dhaka, Bangladesh; 5 Department of Pathology, Dr Sirajul Islam Medical College, Dhaka, Bangladesh; 6 Office of Executive Director (OED), International Centre for Diarrhoeal Disease Research, Bangladesh (icddr,b), Dhaka, Bangladesh; 7 Department of Global Health, University of Washington, Seattle, WA United States of America

**Keywords:** Environmental enteric dysfunction, Maternal undernutrition, Microbiota directed balanced energy protein, Gut microbiota, Pregnancy

## Abstract

**Introduction:**

Studies show, malnourished women of childbearing age with environmental enteric dysfunction (EED) exhibit small intestinal enteropathy resembling that in malnourished children residing in the same community. However, currently there are no universally accepted protocols for validation of these facts. Our current protocol is designed to better understand the mechanism of transmission of the microbiota of mothers with EED to their children perpetuates intergenerational undernutrition. We plan to compare the small intestinal (SI) and fecal microbiota along with plasma, duodenal, and fecal proteomes/ metabolomes and histopathologic evidence of EED in non-pregnant women with and without malnutrition. We also plan to see and compare the effect of microbiota-directed balanced energy protein (MD-BEP) supplementation on these biological parameters between malnourished non-pregnant and pregnant women.

**Methods and analysis:**

This is a community-based intervention study where pregnant women and non-pregnant women of reproductive age (18-35 years) will be screened through household surveys from the Bauniabadh and adjacent slum area of Mirpur, Dhaka (pregnant and non-pregnant cohorts, n=90 each). Using Body Mass Index (BMI), both the groups will be categorized into undernourished (BMI <18.5kg/m2, n=60 each with pregnant and non-pregnant cohort) and well-nourished (BMI >20-24.9 kg/ m2, n=30, each with pregnant and non-pregnant cohort). Upper gastro-intestinal (UGI) endoscopy will be performed on non-pregnant women (both well-nourished and undernourished cohorts) and biopsy samples will be collected for diagnosis of environmental enteric dysfunction (EED) by histopathological scoring. We will compare the small intestinal (SI) and fecal microbiota and the plasma, duodenal, and fecal proteomes/ metabolomes of the undernourished non-pregnant cohort with histopathologic evidence of EED with the well-nourished non-pregnant cohort with no histopathologic evidence of EED (Aim IA). Furthermore, we will perform an intervention study (Aim IB). The undernourished non-pregnant cohort will be randomized into two groups (n=30/arm) and receive daily dietary supplementation with either shelf-stable microbiota-directed balanced energy protein (MD-BEP) or ready-to -use supplementary food balanced energy protein (RUSF-BEP) for 90 days. After cessation of the intervention they will be further followed up for another 270 days and biological samples will be collected at scheduled time points. The well-nourished non-pregnant cohort will not receive any nutritional intervention will serve as a reference control group. The undernourished pregnant cohort will also be randomly (n=30/arm) assigned to receive either MD-BEP or RUSF-BEP daily for 6 months until the child birth and thereafter for 3 months and followed up for another 9 months. During this period anthropometry will be measured and biological samples including fecal and plasma samples will be collected from the mothers and their infants in scheduled time points. Anthropometric, socio-demographic and laboratory assay data will be compared between the groups and candidate EED biomarkers will be correlated with nutritional status, histological analyses and score of EED, plus assessments of microbial community structure will be examined.

**Ethics and dissemination:**

Ethics approval was obtained from the Ethical Review Committee of icddr,b (protocol no: PR-22117; Version 1.2; 29 November 2022). The running approved protocol version is v1.3, dated 07 May 2024. Results of this study will be submitted for publication in peer-reviewed journals.

**Trial registration number:**

ClinicalTrials.gov ID: NCT05862363. Registered on 08 February 2023. https://clinicaltrials.gov/study/NCT05862363.

## Introduction

South Asia bears the highest global burden of undernutrition among women of reproductive age, with prevalence estimates ranging from 10% to 40%. In Bangladesh, the situation is especially concerning, as over 30% of women in this age group are undernourished—among the highest rates observed in developing countries. Maternal undernutrition is a significant determinant of poor birth outcomes, contributing to increased risks of low birth weight, neonatal mortality, and ongoing malnutrition in early childhood. It is estimated to be responsible for approximately 25% to 50% of intrauterine growth restriction. In such a way, nutritional deficits can be passed from mothers to their children, resulting in an intergenerational cycle of undernutrition [1]. In Bangladesh, half of the children under five living in slums experience stunted growth, compared to one-third in non-slum areas [2].

Addressing intergenerational malnutrition—whether through prevention or treatment—remains an urgent and unmet global health priority. Data are scarce on the contribution of the small intestinal (SI) microbiota to pathogenesis, primarily due to the difficulty in obtaining gut biopsy specimens from malnourished individuals. The Bangladesh Environmental Enteric Dysfunction (BEED) study, carried out in the Mirpur urban slum of Dhaka, provided a distinctive opportunity to explore the involvement of the duodenal microbiota in the onset of environmental enteric dysfunction (EED) in children and its link to stunted growth [3]. 110 children, aged 18 ± 2 months, who met the criteria for stunting or being at risk for stunting, failed to respond to nutritional intervention, and for whom consent was obtained, underwent esophagogastro-duodenoscopy (EGD). The BEED study also conducted EGD on thirty-eight malnourished women aged 18–45 years (BMI < 18.5 kg/m²) living in the resource-poor area of Mirpur, who did not respond to an egg, milk, and micronutrient-based nutritional intervention similar to that provided to children [3]. For this study, an operational classification of EED was established based on three histopathologic features observed in hematoxylin and eosin-stained tissue sections: inflammatory cell infiltration in the lamina propria, blunting or atrophy of the small intestinal villi, and crypt hyperplasia or elongation. The results, however, showed no statistically significant correlation between the length-for-age (LAZ) scores of the children.

Interestingly it was observed that 90% of the women had histopathologic evidence of enteropathy. The histopathologic features of their duodenal biopsies mimicked what was documented in the children with EED, including reduction in villus height and number, damage to the small intestinal epithelial barrier, and persistent inflammatory cell infiltration in the lamina propria [4–6]. The study done by *Hossain et al.* revealed that malnourished adults had a significantly higher frequency of subtotal villous atrophy, crypt hyperplasia, and marked cellular infiltration than the healthy controls [5]. Of the 38 women who underwent endoscopy, matched duodenal aspirates, fecal samples, and plasma samples from 22 [BMI 17.5 ± 0.9 kg/m^2^; 26.6 ± 8.2 years old] women were obtained. Matched plasma samples and duodenal biopsy specimens were obtained from 84 out of 110 children. A “core group” of 14 bacterial taxa was identified, each present in at least 80% of the EED-associated aspirate samples [4]. The absolute abundance of 14 shared bacterial strains isolated from children’s duodenal samples exhibiting histopathologic signs of EED and not commonly recognized as enteropathogens - was found to be inversely correlated with linear growth, and positively correlated with components of the expressed duodenal proteome involved in immuno-inflammatory responses.

Based on these findings, in this proposal, we will test the hypothesis that small intestinal microbiota contributes to small intestinal enteropathy and malnutrition in young Bangladeshi women of childbearing age. Based on the hypothesis that transmission of microbiota of mothers with EED to their children perpetuates intergenerational undernutrition, an interventional component will be included involving the administration of a specially designed nutritional supplement aimed at improving gut microbiota in pregnant and non-pregnant low BMI women, termed as the microbiota directed balanced energy protein (MD-BEP).

### Nutritional intervention

Conventional nutritional interventions or low-cost water, sanitation and hygiene (WASH) interventions were found ineffective in reversing EED-related malnutrition [7–9], warranting microbiome-targeted food and other interventions. Recently, nutritional intervention prototypes have been formulated using locally sourced, cost-effective, and culturally appropriate complementary foods in Bangladesh that are commonly consumed. Preclinical tests using gnotobiotic mice and piglets, which were colonized with gut microbiota from Bangladeshi children with acute malnutrition, revealed that these nutritional formulations include elements that increase the presence and positive functions of growth-supporting gut bacteria - bacteria that are usually reduced in these children. Subsequently, multiple microbiota-directed complementary food (MDCF) formulations were evaluated in a preliminary proof-of-concept study involving 12 to 18-month-old children with moderate acute malnutrition (MAM) residing in the Mirpur slum. This controlled, one-month, four-group feeding trial compared three different MDCFs to a standard rice-lentil-based ready-to-use supplementary food (RUSF), which was not designed to influence the gut microbial ecosystem. Among the tested MDCFs, MDCF-2 stood out for its enhanced ability to (i) restore the gut microbiota of MAM-affected children to a composition similar to that of healthy children in the community, and (ii) induce beneficial changes in plasma protein profiles related to metabolism, bone development, immune regulation, and neurodevelopment [10, 11]. These outcomes support the hypothesis that correcting disruptions in gut microbial community development may represent a novel strategy for promoting healthy growth. MDCF-2 is composed of chickpea flour, peanut flour, soy flour, green banana, sugar, soybean oil, and a vitamin-mineral premix. Building on these findings, and in partnership with a major food manufacturer in Bangladesh, a shelf-stable packaged version of MDCF, containing green banana powder, was developed and named the MDCF shelf-stable foil pouch formulation.

The latest World Health Organization (WHO) antenatal care guidelines recommend balanced energy-protein (BEP) supplementation for pregnant women in nutritionally vulnerable populations, aiming to lower the incidence of stillbirths and small-for-gestational-age (SGA) births. Recent meta-analyses further support this recommendation, demonstrating that BEP supplementation during pregnancy significantly improves infant birth weight in undernourished populations [12–14]. In September 2016, the Bill and Melinda Gates Foundation convened an expert consultation to establish nutrient composition guidelines for affordable nutritional supplements intended for pregnant and lactating women in undernourished settings. The consultation reviewed various formulations previously used in BEP supplementation trials and assessed macronutrient and micronutrient requirements based on Dietary Reference Intakes (DRIs) proposed by the U.S. Institute of Medicine (IOM) and FAO/WHO. The panel recommended that women in such contexts receive a daily BEP supplement delivering 250–500 kcal and 14–18 g of protein. For high-risk populations with significant nutritional deficits, the serving size could be increased accordingly. Conversely, in settings with lower nutritional risk, the supplement could provide energy at the lower end of the recommended range [15]. Based on the recommendations, in this study, we developed a modified BEP formulation with the ingredients used in MDCF-2, which is aimed to improve the gut microbiota and are calling it microbiota directed balanced energy protein (MD-BEP). The other arm will receive another modified BEP developed with the ingredients used in RUSF and are calling it ready-to-use supplementary food-balanced energy protein (RUSF-BEP).

### Hypothesis and objectives

Our previous studies suggest that malnourished women of childbearing age living in Mirpur exhibit small intestinal enteropathy resembling that found in Mirpur children with EED [4–6, 18]. In our current study, we will test the hypothesis that SI microbiota contributes to small intestinal enteropathy and malnutrition in young Bangladeshi women of childbearing age. An interventional component will be included involving the administration of MD-BEP or RUSF-BEP in pregnant and non-pregnant undernourished women. This will be based on the hypothesis that transmission of the microbiota of mothers with EED to their children perpetuates intergenerational undernutrition. The overarching goals for the current study will be to (i) delineate mechanisms by which the SI microbial community obtained from undernourished Mirpur women contributes to maternal malnutrition and identify surrogate biomarkers that can be applied to malnourished pregnant women, and (ii) test whether MD-BEP can ameliorate EED as judged by these surrogate endpoints in undernourished women (who are either pregnant or non-pregnant).

### Methods and analysis

**Fig. 1:**
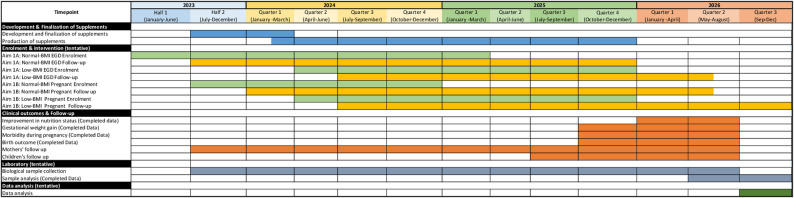
Schedule of study events

This is a community-based intervention study where participants will be enrolled in two cohorts: undernourished pregnant (BMI < 18.5 kg/m^2^; 18–35 years, *n* = 60) and undernourished non-pregnant ((BMI < 18.5 kg/m^2^; 18–35 years, *n* = 60) women from Bauniabadh and adjacent slum areas of Mirpur, Dhaka. For comparison, well-nourished pregnant women (BMI 20–24.9 kg/m^2^; 18–35 years, *n* = 30), who will be screened through household surveys from the Bauniabadh and adjacent slum area of Mirpur, Dhaka, and well-nourished non-pregnant (BMI 20–24.9 kg/m^2^; 18–35 years, *n* = 30) women of reproductive age will be recruited. We will use two specific aims, AIM 1 A and AIM 1B. In AIM 1 A, we will compare the small intestinal (SI) and fecal microbiota and the plasma, duodenal, and fecal proteomes/metabolomes of the undernourished non-pregnant cohort with histopathologic evidence of EED with the well-nourished non-pregnant cohort with no histopathologic evidence of EED undergoing routine endoscopic evaluation for dyspepsia. Upper gastro-intestinal (UGI) endoscopy will be performed on non-pregnant women and biopsy samples will be collected for diagnosis of EED and histopathological scoring. In AIM 1B, we will perform an intervention study. After UGI endoscopy, undernourished non-pregnant cohort will be randomized into two groups (*n* = 30/arm) and receive daily dietary supplementation with either shelf-stable microbiota-directed balanced energy protein (MD-BEP) or ready-to -use supplementary food balanced energy protein (RUSF-BEP) for 90 days. After cessation of the intervention they will be further followed up for another 270 days and biological samples will be collected at scheduled time points. The well-nourished non-pregnant cohort will not receive any nutritional intervention will serve as a reference control group. The undernourished pregnant cohort will also be randomly (*n* = 30/arm) assigned to receive either MD-BEP or RUSF-BEP daily for 6 months until the child birth and thereafter for 3 months and followed up for another 9 months. During this period anthropometry will be measured and biological samples including fecal and plasma samples will be collected from the mothers and their infants in scheduled time points. The well-nourished pregnant cohort will not be supplemented with MD-BEF or RUSF-BEP but will be followed-up with anthropometric measurement and sample collection like undernourished pregnant cohort. Due to ethical concerns related to performing endoscopy in pregnant women, UGI endoscopy in pregnant women will not be performed. Anthropometric, socio-demographic, and laboratory assay data will be compared across study groups. Additionally, potential EED biomarkers will be evaluated for correlations with nutritional status and histopathological findings, alongside analyses of the microbial community structure. Prior to participation, each subject will receive a comprehensive explanation of the study and provide written informed consent. Participants capable of signing will do so, while those unable to sign will provide a thumb impression as an alternative. A separate consent form specific to endoscopy and supplemental interventions will be administered, following the same consent procedure. The endoscopy consent form thoroughly details all aspects of the procedure, including associated risks, ensuring complete transparency. No information pertinent to the participant’s understanding is withheld. While enrolling the neonates, a signed written informed consent from the parents or guardians is obtained. All consents are obtained and documented in presence of the study physician at the study field office in front of a witness, who also sign the document as the witness. This study was conducted and reported in accordance with the CONSORT guidelines. The first participant was enrolled on 28 April 2023. Estimated end date is 31 December 2026. The schedule of study events is presented in Fig. 1.

### AIM 1A: non-pregnant cohort

**Fig. 2:**
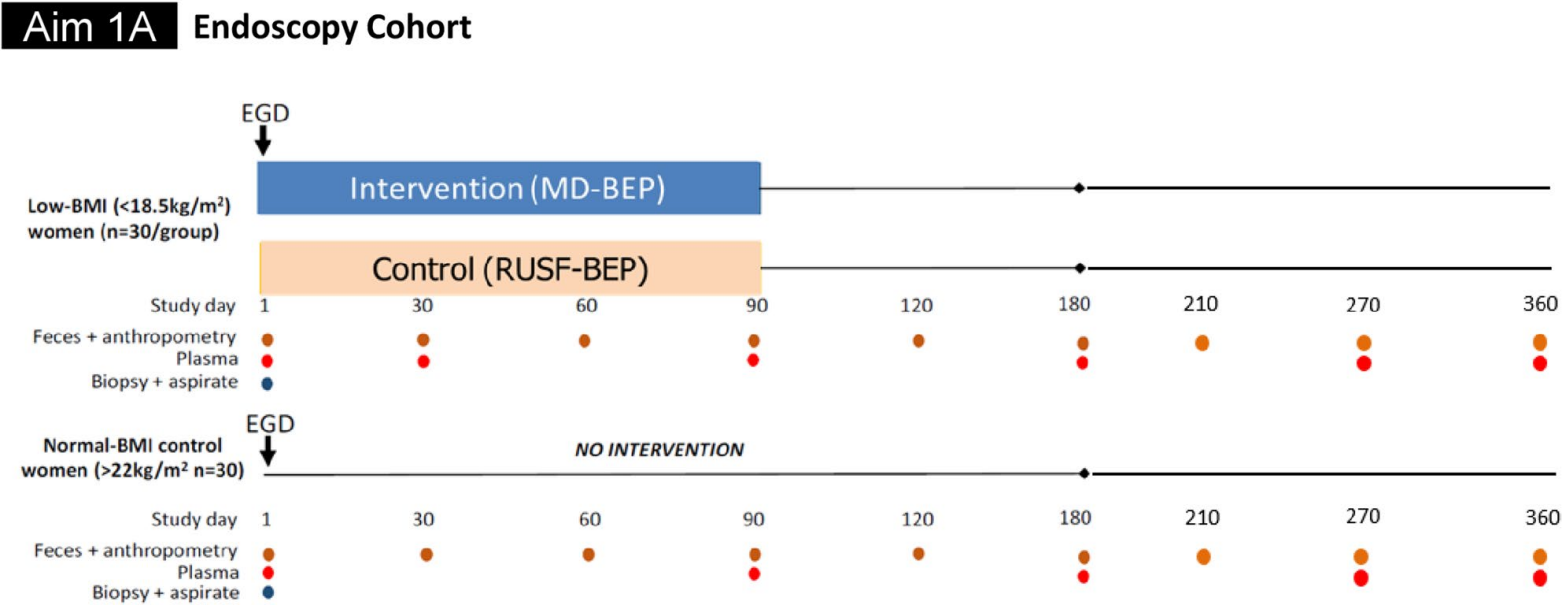
Intervention, sample (plasma, feces, intestinal biopsy and aspirate) collection and follow-up scheme for AIM 1A

**Fig. 3:**
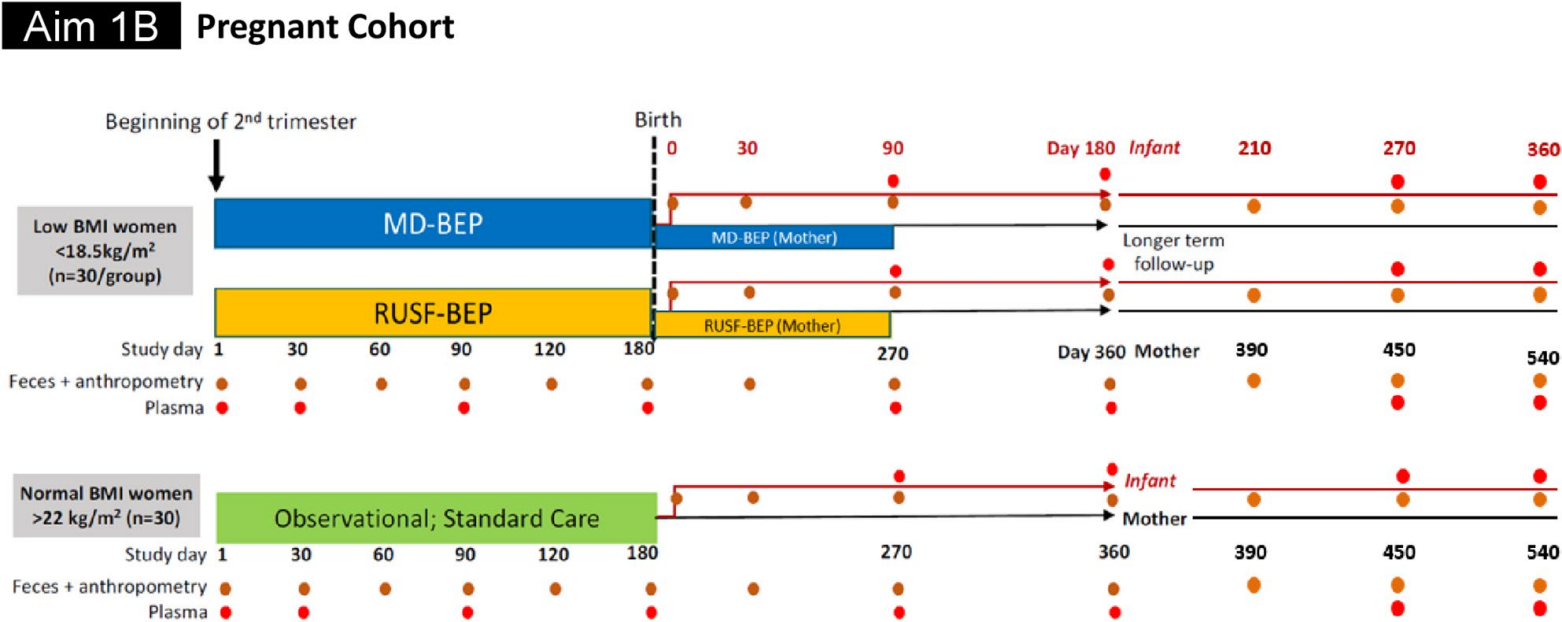
Intervention, sample (plasma, feces, intestinal biopsy and aspirate) collection and follow-up scheme for AIM 1B

In AIM 1 A, for the healthy group, we plan to recruit a cohort of well-nourished Bangladeshi women who will undergo esophagogastroduodenoscopy (EGD) for evaluation of functional dyspepsia and identify 30 well-nourished participants who have normal duodenal mucosal histology. Healthy women (BMI 20–24.9 kg/m^2^) of childbearing age who have been referred for evaluation of functional dyspepsia as defined by Rome IV criteria, will be enrolled from women attending the Gastroenterology OPD of Bangladesh Specialized Hospital (BSH), Dhaka, Bangladesh, and also from the community who meet the inclusion criteria. Duodenal biopsies, duodenal aspirates, plasma and fecal specimens from these participants at the time of endoscopy and serial plasma and fecal samples will be collected according to the follow-up schedule (Fig. 3). These women will be considered as the control group for the non-pregnant cohort and will be followed for 360 days but will not receive any nutritional intervention. Undernourished (< 18.5 kg/m^2^; 18–35 years) women of childbearing age will be enrolled from Bauniabadh and adjacent slum areas of Mirpur, Dhaka, and EGD will be performed among 60 women at icddr, b Dhaka Hospital, or BSH, Dhaka. Participants will be screened through household surveys from the Bauniabadh and adjacent slum area of Mirpur, Dhaka. After EGD, the undernourished women will be randomized into two groups (*n* = 30/arm) and receive daily dietary supplementation with either MD-BEP or RUSF-BEP for a period of 90 days, with a further 270 days of follow-up after cessation of the intervention, and biological samples will be collected from the participants according to the schedule. The EGD scheme is presented in Fig. 2.

### AIM 1B: pregnant cohort

For AIM 1B, beginning at the end of the first trimester, undernourished (< 18.5 kg/m^2^) pregnant women (aged 18–35 years) will be randomly assigned to receive either MD-BEP or RUSF-BEP for the duration of their pregnancy and during the first three postnatal months, in addition to standard antenatal care (*n* = 30/arm). A parallel cohort of age-matched well-nourished pregnant women (*n* = 30) who will not receive any nutritional intervention will serve as a reference control group. Due to safety, sensitivity, and ethical concerns related to performing endoscopy in pregnant women, EGD will not be performed on these women. The women will receive standard antenatal care, will be followed up and serial plasma and fecal samples will be collected according to Fig. 1. These women will be considered as the control group for the pregnant cohort and will not receive any nutritional intervention.

### EGD and biopsy sample collection

Endoscopy will be performed at icddr, b Dhaka Hospital and Bangladesh Specialized Hospital (BSH). Clinical metadata will be collected, including socio-demographic data, dietary history, and use of antibiotics and proton pump inhibitors (PPI) during 12 months prior to EGD. EGD will be performed according to standards recommended by the American College of Gastroenterology (ACG) and the American Society for Gastrointestinal Endoscopy (ASGE) [16]. The endoscopist will use sterile ERCP catheters to collect secretions from the second part of the duodenum (prior to biopsy). Aspirates (~ 1–2 mL/subject) will be divided into aliquots and stored in a manner that preserves bacterial viability using the protocol we employed in the BEED study for children with EED [4].

### Histopathology definition of EED

After EGD, histopathology will be performed to identify women who have EED. An operational categorization of EED used previously based on three histopathologic features (presence of inflammatory cell infiltration in the lamina propria, shortening or loss of small intestinal villi, and increased size or proliferation of the crypts of Lieberkuhn) evident in hematoxylin- and eosin-stained tissue sections will be employed [17]. Histopathologic and immunocytochemical analyses will be conducted at BSH. The biopsies will also be processed at BSH and the standard operating procedure used at BSH will be followed. The scoring systems developed by *Liu et al.* and the categorization of intestinal histomorphology by *Hossain et al.* will be used for the H&E sections [5, 6, 17]. Since inflammatory infiltration typically precedes structural changes like villus atrophy or crypt hyperplasia:

- Grade 0 (No EED): No inflammatory infiltration or structural changes.
- Grade 1 (Mild EED): Inflammatory infiltration in the lamina propria only.
- Grade 2 (Moderate EED): Inflammatory infiltration with either villous blunting/atrophy or crypt hyperplasia.
- Grade 3 (Severe EED): Inflammatory infiltration with both villous and crypt abnormalities.

### Processing of collected biological samples

Schedule for biological sample collection is indicated in Figs. 1 and 2. All biological samples (blood and stool) will be collected in accordance with the standard operating procedures (SOPs) outlined for this protocol. A total of 5 ml of blood will be drawn from each participant. Collected fecal samples will be cryopreserved within 20 min after defecation. At the collection location, the samples will be divided into sterile, pre-labelled 2 mL cryovials and promptly transferred into pre-cooled liquid nitrogen dry shippers for transportation to the laboratory. At the laboratory, vials will be transferred from the dry shipper to a bucket of dry ice which is used during the organization and transfer of samples into 9 × 9 freezer boxes. and immediately placed into a −80 °C freezer. No additives, preservatives or media will be added to the fecal samples. The preparation and transport of all biological samples will strictly adhere to the biosafety laws and procedures of both institution’s and international standards. An aliquot of each fecal sample will be used at icddr, b for (i) Taqman Array Card (TAC)-based assays of 34 enteropathogens and (ii) commonly used clinical markers of intestinal inflammation and barrier disruption [ELISA of several fecal (and plasma) biomarkers including CRP, AGP, myeloperoxidase, neopterin, alpha-1 anti-trypsin, calprotectin, Reg1B, lipocalin-2 (LCN2), and Duox-2].

### Details of intervention products

#### Microbiota directed- balanced energy protein (MD-BEP)

Every woman in the MD-BEP arm will be offered a single serving of MD-BEP daily, aimed as a supplement to the usual diet received by these women. The feeding will initially take place in the field office under direct supervision for at least one week, and continued at home afterwards. The feeding will be supervised by the respective field research assistants (FRAs) in regular intervals at home. MD-BEP will provide approximately 500 Kcal, 14 g of protein and the necessary micronutrients, and the substrate for the beneficial microbiota proliferation.

#### Ready-to-use supplementary food- balanced energy protein (RUSF-BEP)

Women in the RUSF-BEP group will receive a single serving of RUSF-BEP daily, similar to the MD-BEP arm. The feeding will initially in the field office for at least one week, and continued at home afterwards. The feeding will be supervised by the respective FRAs. Each serving of RUSF-BEP will also provide approximately 500 Kcal, 14 g of protein, and necessary micronutrients following the recommendations from the expert panel convened by the BMGF on nutritional supplementation in pregnancy [15].

The amount in each serving of the supplements and the compositions are shown in Tables 1 and 2, respectively, which are in conformity with the recommendations from the expert panel convened by the BMGF on nutritional supplementation in pregnancy [15].

**Table 1:** Ingredients used in MD-BEP and RUSF-BEP formulations with amount per serving

Ingredients	MD-BEP (gms)	RUSF-BEP (gms)
Chickpea, flour	14.00	-
Soy flour	18.50	-
Peanut, paste	14.25	-
Green banana powder	12.50	-
Sugar, powder	11.90	10.30
Soybean Oil	20.00	28.50
Micronutrient Premix	2.30	2.30
Amino acid powder (Lysine 0.15, Methionine 0.05, Cysteine 0.05)	0.25	-
Stabilizers (Lecithin)	0.30	0.30
Rice, flour	-	13.40
Lentil, flour	-	14.60
Skimmed Milk, Powder	-	27.60
Total	94	97

Enrolled women will be closely monitored by Field Research Assistants for one week to identify any potential side effects or adverse events (e.g., rash, urticaria due to food allergy, or notable changes in clinical status). Any such events will be managed in accordance with the standard of care. Study participants will be given dietary counseling focused on promoting dietary diversity, incorporating vegetable oil into cooked meals as an energy source, and encouraging regular consumption of animal-based foods such as small fish or chicken, alongside a consistent intake of vegetables. The study participants will also receive support for any intercurrent illnesses detected by the study team as well as proper referral will be provided.

**Table 2:** Nutritional composition of MD-BEP and RUSF-BEP

Nutritional composition	BEP recommendation	MD-BEP (per 94 g)	RUSF-BEP (per 97 g)
Total Energy(kcal)	250–500	494.62	497.54
Protein (g)	14–18	14.18	14.33
Energy from Added Sugar, (% of total energy)	< 10%	9.36%	8.05%
Energy from Fat (FER), (% of total energy)	10–60%	58.48%	53.08%
Total ω−3 (g)	≥ 1.30	1.70	2.01
(ω−6/ω−3) ratio	5–10:1	8.29:1	7.34:1
Total Trans-fatty acid (g)	< 1.00	0.16	0.19
DIAAS	≥ 90%	91.67%	123.64%

#### Manufacturing of intervention products

The servings will be provided in sterilized formulations packaged in trilaminar foil pouches developed in Natore Agro Limited (NAL) factory, a sister concern of a renowned food manufacturing company of Bangladesh, PRAN. A standardized production process will be followed to maintain the quality of the formulations throughout the project period. All raw materials will first undergo a rigorous quality control process. The selected ingredients will then be roasted in a temperature-controlled hot air oven and ground into a fine powder or paste using a heavy-duty electric grinder, as appropriate. The mixture will be portioned into trilaminar foil pouches, with each processing step closely monitored. Pouches will be sealed using an impulse sealer to ensure airtight packaging, followed by sterilization in a retort machine at 105 °C and 0.123 MPa for 10 min. This sterilization protocol is designed to extend shelf life and maintain the food’s organoleptic qualities. Prior to finalization, supplement prototypes will be assessed for microbiological safety and sensory acceptability. Additionally, during manufacturing and distribution, random samples from each batch will be tested for microbial contamination and organoleptic characteristics.

### Data collection

At study initiation, data on demographic characteristics—such as household wealth, husband’s occupation and income, housing conditions, family structure, and home environment—will be collected. Morbidity information will be gathered on a weekly basis throughout the intervention period.

### Anthropometry and body composition

Nutritional status will be assessed through anthropometry. Body weight and BMI will be determined at the anthropometry timepoints mentioned in Figs. 2 and 3. Bioelectric Impedance Analysis (BIA) will be used to measure total fat and fat-free mass before, during and after the intervention.

### Follow-up of pregnant women

Pregnant women will be enrolled at the beginning of second trimester and followed up till one year after childbirth. For the undernourished pregnant women, antenatal care (ANC) services from nearby healthcare facilities will be ensured by study staff. Trained Health Workers (HWs) will visit the homes of all enrolled pregnant women once a month and will involve the family members, including mothers, mothers-in-law, and husbands, along with the pregnant women in the sessions according to the convenience and availability of the participant and her family members. The woman and her family will receive advice on the importance of adequate weight gain during pregnancy, additional energy requirements, and improving dietary diversity. The pregnant women will be advised to seek routine ANC according to the schedule and to regularly take micronutrients (iron, folic acid, and calcium) provided through routine ANC. On each visit, the participants’ dietary diversity and rate of weight gain will be evaluated, and they will be given instant feedback. The participants will have access to field offices where they shall receive a thorough physical check-up by a registered physician. If any unwanted event occurs during the follow-up period, the participants will be referred to the nearest maternal clinic or appropriate healthcare facility.

### Available health care services for low-BMI pregnant women in the study site

The Bauniabadh area of Dhaka city is located in ward-5 of Dhaka North City Corporation [3, 18, 19]. The accessibility of various facilities and services for secondary and tertiary healthcare has increased significantly. Medical clinics and diagnostic centers are now widely available across the region. Medical clinics and diagnostic centers have become numerous all over the area outside slums. In this area, health infrastructure facilities, managed and operated by various organizations, can be classified into three categories: (i) public, (ii) private, and (iii) non-governmental organizations (NGOs). Currently, in this particular area, primary health care targeting maternal and child nutrition is provided by The Asian Development Bank (ADB)-supported Urban Primary Health Care Project (UPHCP II) and the USAID-supported NGO Services Delivery Program, which served as a precursor to the current Smiling Sun Franchise Program (SSFP), Urban Health Center by BRAC, and Radda MCH-FP center. The services provided by these facilities include basic medical consultation, diagnostics, EPI, pregnancy and delivery services, including ANC, Infant and Young Child Feeding (IYCF) services, and nutrition counseling.

### Sample size calculation and outcome

For normal-BMI (BMI 20–24.9 kg/m^2^) women of childbearing age or healthy volunteers undergoing EGD, the goal is to identify 30 normal-BMI participants who will undergo EGD for evaluation of functional dyspepsia and will have normal duodenal mucosal histology. We plan to recruit 100 individuals for EGD, and anticipate that 60 will have duodenal mucosa without macroscopic evidence of active acid-peptic disease, of whom 30 will have normal duodenal mucosal histology. These calculations are based on the team’s gastroenterologist who has 15-year experience with > 30,000 subjects from this socioeconomic group and geographic locale. These specimens from normal BMI participants without enteropathy will be compared with those of 60 undernourished low-BMI (< 18.5 kg/m2) women of childbearing age. As this is an experimental study, no formal power calculation has been performed for this exploratory research study. The same applies for the pregnant cohort.

### Data analysis

The cohort-specific characteristics of the recruited participants will be described by exploratory data analysis. Normally distributed variables will be summarized as mean and standard deviation, whereas, median and inter-quartile range will be used for the variables following skewed distributions. Binary and categorical variables will be presented as counts and percentages. Appropriate statistical tests (Student’s t-tests, Pearson chi-square tests, and Mann-Whitney test) will be used to detect the differences in baseline characteristics of the two intervention groups. A probability of < 0.05 will be considered statistically significant. The correlation between the confounders and outcome will be evaluated using Pearson or Spearman correlation, whichever is appropriate. Multi-collinearity between the predictor variables will be assessed using variance inflation factor (VIF), and a predefined cut-off of 2 will indicate the absence of multi-collinearity.

#### Modeling the clinical effectiveness of the intervention

A linear mixed effects model—a statistical technique for analyzing longitudinal data—will be employed to assess the impact of the intervention on the outcomes described above. The model for continuous outcome variables is specified as:

$$Yi=Xi\beta+Zibi+\varepsilon i,$$

where Yi represents the vector of responses for each subject across multiple follow-up time points. Xi denotes the matrix of fixed effects (such as treatment, baseline covariates, and general time trends) with associated regression coefficients β. Zi represents the matrix of random effects accounting for subject-specific trends (e.g., morbidity, adherence to the intervention), with corresponding random effect coefficients bi, and εi denotes the vector of residual errors. Both intention-to-treat and per-protocol analyses will be conducted.

#### Modeling the changes in centiles of anthropometry curves

Percentage changes in weight and BMI will be calculated by subtracting baseline values from follow-up measurements, dividing the difference by the baseline value, and then multiplying by 100. Generalized linear mixed-effects models (GLMMs) will be used to model the percent changes of indices. GLMMs will estimate the probability of improvement in the BMI and weight in the intervention group from baseline in comparison to the control group, after adjusting for all the other possible covariates.

### Data safety monitoring plan (DSMP)

Data collection tools for this study will include case report forms, laboratory worksheets, and source documents. Comprehensive source documentation—such as study visit records and laboratory reports—will be maintained for each participant in individual study files. All biological samples will be collected in accordance with Good Clinical Practice (GCP) guidelines. To protect participant confidentiality and ensure traceability, all laboratory specimens, reports, data collection forms, and administrative documents will be labeled with coded identifiers. Study-related documents will be securely stored in locked cabinets within restricted-access rooms. The electronic database, maintained at icddr, b, will be password-protected and accessible only to authorized members of the research team. Data entry and cleaning will also be carried out at icddr, b.

### Adverse events

Anticipated adverse events (AEs) under this protocol include those associated with endoscopy and biopsy procedures, pregnancy and childbirth, and phlebotomy. Both serious and non-serious AEs will be evaluated in terms of severity, their potential relationship to study participation, actions taken in response, and eventual outcomes. All serious adverse events (SAEs) are reported to the Ethical Review Committee (ERC) of icddr, b within 24 h of the site becoming aware of the incident. If medical care outside the study protocol is needed, all necessary and available treatments will be provided, or appropriate referrals will be arranged to ensure adequate care.

## Data Availability

No datasets were generated or analysed during the current study. All relevant data from this study will be made available upon study completion.
